# Yeast Models and Molecular Mechanisms of Neurodegenerative Diseases

**DOI:** 10.3390/ijms22168775

**Published:** 2021-08-16

**Authors:** Joanna Kaminska, Teresa Zoladek

**Affiliations:** Institute of Biochemistry and Biophysics PAS, 02-106 Warsaw, Poland

Neurodegenerative diseases are a group of age-related diseases and a growing problem in an aging society. In addition to major neurodegenerative diseases, such as Alzheimer’s, Parkinson’s, and Huntington’s diseases, there are numerous rare and even ultra-rare ones. These diseases are a consequence of diverse pathological genetic and physiological alterations in cells but manifest by common dysfunctions of the central or peripheral nervous systems. Interestingly, in a group of genes linked to neurodegenerative diseases, the genes affecting mitochondria are overrepresented [1]. Moreover, even though the primary mutation does not affect mitochondria directly, the mitochondria are often disturbed. This probably indicates that because of high demand for ATP, mitochondria are crucial for neurons. In the group of genes affecting the mitochondria are also those that code for proteins responsible for the formation or functioning of membrane contact sites (MCSs) between the mitochondria and other organelles [2,3,4,5]. MCSs are sites of inter-organellar communication with various functions, such as lipid and metabolite exchange, organelle dynamics, positioning, distribution, and clearance. MCSs between mitochondria and the endoplasmic reticulum (ER) are also especially important for maintaining calcium ion (Ca^2+^) homeostasis. Thus, mitochondrial MCSs disturbance affects mitochondrial biogenesis and functions.

The Special Issue of the International Journal of Molecular Sciences (MDPI) entitled Yeast Models and Molecular Mechanisms of Neurodegenerative Diseases presents advances in the research on the pathogenesis of a subset of neurological diseases, made using a yeast model. It includes diseases (1) caused by mutations in the four *VPS13* genes (*VPS13A-D*), such as chorea-acanthocytosis, Cohen syndrome, early onset Parkinson’s disease, and spastic ataxia/paraplegia; (2) associated with the formation of inclusions containing TDP-43 protein, such as amyotrophic lateral sclerosis (ALS); (3) associated with the formation of Lewy bodies, such as Parkinson’s disease; (4) associated with mutations in mitochondrial DNA (mtDNA); and (5) caused by mutations in 1 of over 100 different genes linked to pathologies of the peripheral nervous system, i.e., the Charcot–Marie–Tooth (CMT)-type diseases.

The *VPS13A-D* genes encode the four human *VPS13A-D* homologous proteins localized to different subsets of MCSs [6]. This diverse localization could explain why different *VPS13* gene mutations are responsible for distinct neurological disorders. Moreover, *VPS13A* and *VPS13D* proteins link mitochondria to the ER and, therefore, are another example of proteins whose deficiencies cause mitochondrial dysfunction, leading to neurological diseases. *VPS13* proteins are responsible for lipid transport between membranes [7,8]. However, how this function is relevant to diseases is still unknown. Dziurdzik and Conibear [9] showed the progress made from the study of the yeast *Vps13* protein in understanding the determinants responsible for the proper localization of *VPS13* proteins in a cell, especially recruitment to multiple MCSs. It is important to find the basis of *VPS13* interaction with different organelles and know the precise function of each homologue at specific MCSs to understand the pathogenesis of *VPS13*-related diseases.

Soczewka et al. [10] and Wardaszka et al. [11] further characterized the importance of *VPS13* proteins for cell physiology by studying the yeast *vps13*∆ mutant devoid of the yeast *Vps13* protein. A simple phenotype, i.e., the hypersensitivity of the *vps13*∆ mutant to the commonly used detergent sodium dodecyl sulphate (SDS), allowed the authors to use the power of yeast genetics to discover changes in copper homeostasis, in addition to the already described changes in calcium [12] and iron [13] homeostasis. Moreover, they showed several ways to alleviate the growth defect caused by the *vps13*∆ mutant. The first way shown in this Special Issue is overexpression of the *RCN2* gene, encoding the Rcn2 protein, which causes partial downregulation of calcineurin activity, a key calcium-dependent phosphatase, by inhibiting only the subset of catalytic complexes [11]. The second way is the increase in the cellular level of copper ions via simple supplementation with copper salts, treatment with copper ionophores, or genetic manipulation, resulting in an increase in copper uptake [10]. This is an interesting finding since copper ion homeostasis has already been shown to be affected in Alzheimer’s and Parkinson’s diseases and copper ionophores are in clinical trials [14]. These results point to calcineurin and copper homeostasis as potential therapeutic targets suitable for testing in higher eukaryotic models of *VPS13*-related diseases.

Two other papers focused on diseases characterized by aggregate formation. Malcova et al. [15] described modeling of stress granule (SG) formation upon heat shock. SGs contain components of translational machinery, are sites of mRNA sequestration, and help cells adapt to stress. Using this model, the authors showed a high propensity for the accumulation of the mutant Rpg1-3 protein, a subunit of the translation initiation factor eIF3a, in SGs. Interestingly, the authors documented that these SGs are associated with MCSs, called ERMES, formed between the ER and mitochondria. SGs containing Rpg1-3 have been characterized; they are formed at a lower temperature than those containing wild-type Rpg1 and are more stable. Interestingly, the delayed disassembly of these SGs is reversed by the overproduction of TDP-43, which is a human protein associated with ALS, a disease connected with the dysfunction of mitochondrion-ER contacts (MERCs) [16]. Disease-causing amino acid residue substitutions in TDP-43 result in the sequestration of the mutant TDP-43 protein, together with its interacting partners, in cytoplasmic aggregates [16]. These aggregates and SGs could be cytotoxic and contribute to the death of neurons [17]. Based on the results, the authors proposed the use of an Rpg1-labeled SG variant as a model to study the function of TDP-43. Further studies will determine the impact of ERMES on SG formation and TDP-43 action.

In another study, Seynnaeve et al. [18] showed the relevance of yeasts, especially *S. cerevisiae*, as a model to unravel mechanisms contributing to Synphilin-1-dependent pathology in the context of neurodegenerative diseases, such as Parkinson’s disease and Lewy body dementia. They focused on understanding the influence of sugar metabolism disturbances promoting the glycation of proteins on the aggregation of Synphilin-1, which is a partner of α-Synuclein, a primary constituent of Lewy bodies. They studied the effects of a disrupted glyoxalase system and aldose reductase function, due to the lack of the enzymes Glo1, Glo2, and Gre3, on Synphilin-1 inclusion formation and cell growth. The data showed that the expression of the human *SNCAIP* gene, encoding Synphilin-1, in *S. cerevisiae* cells lacking Glo2 or Gre3 activity increases oxidative stress, causes the formation of large Synphilin-1 inclusions, and inhibits growth. In yeast, different structures are responsible for aggregate sequestration, such as cytoplasmic quality control compartment (CytoQ), juxta/intranuclear quality control compartment (JUNQ/INQ), and insoluble protein deposit (IPOD) [19,20]. The Synphilin-1 aggregates were localized to JUNQ and IPOD in wild-type cells but in 1-3 IPODs in *glo2*Δ and *gre3*Δ mutant cells. Interestingly, in a previous study, the inclusions that were shown to be in IPODs were associated with the MCSs called the mitochondria–vacuole patch (v-CLAMP), linking mitochondria and vacuoles, the yeast counterparts of lysosomes [21]. It would be interesting to know whether the lack of v-CLAMP influences Synphilin-1 inclusion formation and cytotoxicity in wild-type cells and in cells lacking a glyoxalase system or aldose reductase.

Mutations in mitochondrial DNA (mtDNA), in the *ATP6* gene encoding subunit *a*/Atp6 of ATP synthase, cause several diseases, including neurogenic ataxia, retinitis pigmentosa (NARP), and maternally inherited Leigh syndrome (MILS). However, it is difficult to determine the pathogenicity and consequences of the mtDNA mutations found in the cells of patients with these lesions due to heteroplasmy of mtDNA. This can be conducted more easily in yeast cells, which are homoplasmic and, importantly, have the ability to ferment glucose and survive oxidative phosphorylation defects caused by loss-of-function mtDNA mutations [22]. One such mutation is in the m.9191T>C position; this mutation causes substitution of evolutionarily conserved leucine 222 for the proline residue, and leads to MILS. Su et al. [23] studied in yeast, the effect of intragenic suppressor mutations of *atp6-L242P,* the mutation equivalent to m.9191T>C, on the functioning of ATP synthase at the molecular level. The experimental studies and structural modeling of mutant *a* subunit together with a ring of ten *c* subunits allowed the authors to propose that leucine 242 is critical for optimal proton conduction through the membrane domain of ATP synthase and that the pharmacological targeting approach is difficult for patients with m.9191T>C mutations since the elimination of proline from 222 position is the only way to recover the bioenergetics capacity.

Although yeast is extensively used to study the effects of mtDNA mutations, the use of yeast as a model to study CMT-type diseases has only recently begun. These rare diseases are characterized by similar clinical symptoms but extremely high genetic heterogeneity. There is an urgent need to develop new models for this group of diseases. Rzepnikowska et al. [24] reviewed the research already performed on some CMT-type diseases using a yeast model, mostly focusing on discrimination between pathogenic and non-pathogenic variants. The authors also postulated using yeast to study the mechanisms of pathology and to discover new therapies for CMT-type diseases. It is possible to do this even when there is no yeast gene homologous to that mutated in humans. Such a strategy was successfully employed for major neurodegenerative diseases, such as Alzheimer’s and Parkinson’s diseases. Yeast model studies promise great advancements in understanding the pathogenesis of CMT-type disease in the future.

To conclude, we were pleased to guest-edit this Special Issue, the aim of which was to collect relevant papers reflecting the increasing interest in rare and ultra-rare neurodegenerative diseases. We hope this issue will reach a wide audience in the scientific community and boost further research to obtain new insights into the molecular pathogenesis of these diseases and the development of new therapeutic strategies.
